# The Role of MaWRKY70 in Regulating Lipoxygenase Gene Transcription during Chilling Injury Development in Banana Fruit

**DOI:** 10.3390/foods13060854

**Published:** 2024-03-11

**Authors:** Han Lin, Lijuan Bai, Wei Wei, Wenbing Su, Yanting Wu, Rong Wu, Hui Wang, Jianye Chen, Hetong Lin, Zhongqi Fan

**Affiliations:** 1 Institute of Postharvest Technology of Agricultural Products, College of Food Science, Fujian Agriculture and Forestry University, Fuzhou 350002, China; linhan_haha@163.com (H.L.); bailijuan2024@163.com (L.B.); wuyanting2024@163.com (Y.W.); 15093706039@163.com (R.W.); xiafengxixi@126.com (H.W.); hetonglin@163.com (H.L.); 2 Fruit Research Institute, Fujian Academy of Agricultural Science, Fuzhou 350013, China; suwenbing13@163.com; 3 Guangdong Provincial Key Laboratory of Postharvest Science of Fruits and Vegetables, College of Horticulture, South China Agricultural University, Guangzhou 510642, China; weiwei@scau.edu.cn (W.W.); chenjianye@scau.edu.cn (J.C.)

**Keywords:** banana, chilling injury, WRKY, LOX, transcriptional regulation

## Abstract

Banana is a typical cold-sensitive fruit; it is prone to chilling injury (CI), resulting in a quality deterioration and commodity reduction. However, the molecular mechanism underlying CI development is unclear. In this study, cold storage (7 °C for 5 days) was used to induce CI symptoms in bananas. As compared with the control storage (22 °C for 5 days), cold storage increased the CI index and cell membrane permeability. Moreover, we found that the expression levels of the WRKY transcription factor *MaWRKY70* were increased consistently with the progression of CI development. A subcellular localization assay revealed that MaWRKY70 was localized in the nucleus. Transcriptional activation analyses showed that MaWRKY70 processed a transactivation ability. Further, an electrophoretic mobility shift assay (EMSA) and dual-luciferase reporter (DLR) assays showed that MaWRKY70 was directly bound to the W-box motifs in the promoters of four lipoxygenase (LOX) genes associated with membrane lipid degradation and activated their transcription. Collectively, these findings demonstrate that MaWRKY70 activates the transcription of *MaLOXs*, thereby acting as a possible positive modulator of postharvest CI development in banana fruit.

## 1. Introduction

Banana (*Musa acuminata*) is one of the most widely grown fruits in tropical and subtropical regions due to its sweet flavor and high nutritional value [1,2]. However, banana is a typical cold-sensitive fruit that is susceptible to chilling injury (CI) symptoms, including pitting, water soaking, and even abnormal fruit ripening, leading to a quality deterioration and commodity loss [3,4]. Therefore, it is important to investigate the underlying molecular mechanism of CI development in postharvest banana fruit.

Plants are regularly exposed to changing environments throughout their life cycle and respond and acclimate to this environmental stress to survive and reproduce [5,6]. Sophisticated signaling cascades are used to induce changes in the expression of temperature-responsive genes that enable plants to withstand temperature stress [7]. The alterations of plant membrane function and structure caused by cold are considered to be the principal events of CI [8]. Under cold stress, the liquid crystal of the cell membrane undergoes a phase transition and transforms into a gel state with poor fluidity. This phase transition leads to an increase in membrane permeability, ultimately resulting in physiology disorder, membrane damage, and CI occurrence [9]. Membrane lipids are the predominant components of plant cell membranes; therefore, the composition of the membrane lipids has an important role in maintaining the stability of the membrane’s structure and function [10]. The composition and content of membrane lipids are regulated by a series of enzymes, such as phospholipase A (PLA), phospholipase D (PLD), lipoxygenase (LOX), and lipase. Specifically, phospholipids are degraded by PLD, PLA, and lipase to free fatty acids (FFAs). LOX promotes membrane lipids’ peroxidation via catalyzing the conversion of unsaturated fatty acids (USFAs) to saturated fatty acids (SFAs) [11]. Previous reports have shown that the enzymatic activity and the transcription of PLA, PLD, LOX, and lipase in banana fruit were induced by a cold environment during storage [4]. Moreover, the enzyme activity and gene expression of PLD and LOX increased in cold-stored green bell peppers [12]. The activity of PLD and LOX rose in line with CI development in zucchini fruit [13]. Generally, the occurrence of CI is caused by changes in membrane lipid degradation and peroxidation enzyme activity in fresh fruit.

WRKYs are one of the largest transcription factor (TF) families in plants. It is widely known that WRKYs are essential modulators of biotic and abiotic stress [14]. In 1994, Ishiguro and Nakamura cloned the first WRKY transcription factor, SPF1, from sweet potatoes. In recent years, with the rapid development of molecular biology and bioinformatics techniques, as well as the unveiling of numerous plant genome sequences, a large number of *WRKY* genes have been discovered and identified from plants, such as Arabidopsis, corn, rice, potato, tomato, cotton, and cucumber [15]. For example, the overexpression of the *OsWRKY71* and *OsWRKY76* genes in rice enhances its cold resistance, while the overexpression of *OsWRKY63* in rice exacerbates the membrane damage caused by low temperatures, leading to a cold tolerance decrease in the plant. The *oswrky63* mutant plant improved its cold tolerance [16,17]. *Vitis amurensis VaWRKY12* was upregulated by cold and the overexpression of *VaWRKY12* in Arabidopsis could enhance its cold tolerance [18]. The overexpression *KoWRKY40* from *Kandelia obovate* in Arabidopsis improved its cold resistance by regulating the antioxidant system and cold signaling pathway (ICE-CBF-COR) [19]. The overexpression of *PmWKY57* from *Prunus mume* reduced the cold sensitivity in *Arabidopsis* by upregulating the expression of cold-response genes, including *AtCOR6.6*, *AtCOR47*, *AtKIN1*, and *AtRCI2A* [20]. To date, however, little has been reported about the molecular aspects of cold-induced WRKY TFs in cold-sensitive fruit, such as banana.

Previously, we demonstrated that CI development in banana fruit was caused by membrane integrity damage and was associated with the enzymatic and genetic manipulation of membrane lipid metabolism. These activities facilitated the degradation of membrane phospholipids and USFAs in banana fruit during the cold storage period [4]. Nevertheless, the transcriptional regulatory mechanisms underpinning WRKY-TF-modulated membrane lipid metabolism in cold-stored banana fruit remain largely unknown. Here, a cold-induced WRKY TF, MaWRKY70, was isolated and characterized from banana fruit. We demonstrate that MaWRKY70 is positively associated with CI development in postharvest banana fruit by activating four *LOX* genes, namely *MaLOX1*, *MaLOX3.1*, *MaLOX3.2* and *MaLOX4*. Our findings thus provide a novel link between MaWRKY70 and CI development in banana fruit. 

## 2. Materials and Methods

### 2.1. Plant Material and Treatments

Banana fruits at 70–80% maturation were taken from an orchard in Zhangzhou, Fujian, China. The banana fruits were separated into individual fingers and selected based on the absence of mechanical damage and disease, with a uniform size and color. The selected bananas were washed in water and then soaked in prochloraz solution for one minute. After air drying, the fruits were divided randomly into two groups. One group was stored at 7 °C (cold treatment); the other group was stored at 22 °C (control). Banana peel tissues were sampled at 0, 1, 2, 3, 4, and 5 d; frozen in liquid nitrogen; and preserved at −80 °C for further use [4].

### 2.2. Assessment of CI Index and Cell Membrane Permeability

The CI index was assessed following the methodology of Li et al. [4]. Banana fruits are graded for cold damage according to the size of external browning areas, where 0 indicates no browning area; 1 indicates areas with 1–25% browning; 2 indicates areas with 25–50% browning; 3 indicates areas with 50–75% browning; and 4 indicates areas with 75–100% browning [21,22].

Cell membrane permeability was determined by using the method of Li et al. [4]; the results were calculated as the ratio of the electrical conductivity in both the non-boiled and boiled state and were expressed in %.

### 2.3. RNA Extraction and cDNA Synthesis

The total RNA of banana peel was extracted by an RNAprep Pure Plant Kit (Tiangen, Bejing, China). The protocol of RNA extraction followed the manufacturer’s instructions, including the following steps: (a) 100 mg fruit tissues were ground into a powder in liquid nitrogen; 450 μL buffer RL was added (β-mercaptoethanol was added before use); and it was vortexed vigorously, shaken, and stirred evenly; (b) all solutions were transferred to the RNAase-free filter column set (CS) and centrifuged at 13,400× *g* for 5 min; the supernatant was aspirated from the collection tube into the RNase-free centrifuge tube, avoiding contact with the cell debris precipitate in the collection tube; (c) 225 μL anhydrous ethanol was added and the obtained solution and precipitation were transferred together into the adsorption column set (CR3); it was centrifuged at 13,400× *g* for 60 s; the waste liquid was discarded from the collection tube, and the adsorption column CR3 was replaced in the collection tube; (d) 350 μL deproteinized solution RW1 was added to the adsorption column CR3 and it was then centrifuged at 13,400× *g* for 60 s; the waste liquid was discarded from the collection tube and the adsorption column CR3 was replaced in the collection tube; (e) 80 μL DNaseI working solution was added to the center of the adsorption column CR3 and placed at room temperature for 15 min; (f) 350 μL deproteinized solution RW1 was added to the adsorption column CR3 and it was then centrifuged at 13,400× *g* for 60 s; (g) 500 μL RW buffer was added (ethyl alcohol was added before use) and it was placed at room temperature for 2 min and then centrifuged at 13,400× *g* for 60 s; the waste liquid was discarded from the collection tube and the adsorption column CR3 was placed into the collection tube; this was repeat once; (h) it was centrifuged at 13,400× *g* for 2 min, the waste liquid was discarded from the collection tube, and it was placed at room temperature for 5 min; (i) the adsorption column CR3 was placed into a new RNase-free centrifuge tube and 50 μL RNase-free ddH_2_O was added; it was placed at room temperature for 2 min and centrifuged at 13,400× *g* for 2 min to obtain an RNA solution.

First-strand cDNA was synthesized via reverse transcription by a cDNA synthesis kit (Vazyme, Nanjing, China), following the manufacturer’s instructions, including the following steps: (a) the genomic DNA was removed and RNase-free ddH_2_O was added, with 4 × gDNA wiper mix and the template RNA; the reaction took place at 42 °C for 2 min; (b) to prepare the reverse transcription reaction system, 5 × HiScript III qRT SuperMix was added to the above solution; (c) the reverse transcription reaction was performed at 37 °C for 15 min and 85 °C for 5 s.

RT-qPCR was carried out using the Taq Pro universal SYBR qPCR Master Mix (Vazyme, China) on a Bio-Rad CFX96 Real-Time PCR System. The required primers were designed using the Integrated DNA Technologies Website (https://sg.idtdna.com/pages) (accessed on 10 June 2022), and all primers are recorded in Table S1. *MaEIF5A-2* was used as the reference gene [23]. 

### 2.4. Subcellular Localization

The full-length MaWRKY70 was cloned into the pBE-GFP vector. The pBE-GFP-MaWRKY70 or pBE-GFP (positive control) was transferred to the *Agrobacterium tumefaciens* strain EHA105 and then injected into tobacco leaves. NLS-mCherry was used as the nuclear marker. After 48 h of infiltration, the fluorescent signal of GFP was observed and imaged through a fluorescence microscope (ZEISS, Oberkochen, Germany) [24,25]. 

### 2.5. Dual-Luciferase Transient Expression Analysis

For the analyses of MaWRKY70 transactivation, its coding sequence was inserted into the pGreenII BD-62-SK vector as an effector. The pGreenII 0800-LUC vector, which contains firefly luciferase (LUC) and renilla luciferase (REN), was used as the reporter. For the analyses of MaWRKY70 regarding the activation of the *MaLOX1*, *MaLOX3.1*, *MaLOX3.2*, and *MaLOX4* promoters, the MaWRKY70 coding region was cloned into the pGreenII 62-SK vector as an effector, while the promoters of *MaLOX1*, *MaLOX3.1*, *MaLOX3.2*, and *MaLOX4* were cloned into the pGreenII 0800-LUC vector as a reporter. Different pairs of effectors and reporters were co-transferred to tobacco leaves. After 48 h of infiltration, the LUC and REN activity was measured by a Dual Luciferase Assay Kit (Promega, Madison, WI, USA) in a Luminoskan Ascent Microplate Luminometer (Thermo, Waltham, MA, USA). The LUC/REN ratio was calculated to obtain results on the transcriptional activity [26]. 

### 2.6. Y2H Assay

To estimate the transcriptional activity of MaWRKY70 in yeast, the full-length MaWRKY70 was inserted into the pGBK7 vector. The pGBK7-MaWRKY70, pGBK7-empty, and pGBK7-p53+pGAD7 plasmids were transformed into the yeast strain Y2H, respectively, following growth on synthetic defined (SD) medium lacking tryptophan (SD/-Trp) or tryptophan, histidine, and adenine (SD/-Trp-His-Ade). The transcriptional activity of MaWRKY70 was determined according to the growth state and *α*-galactosidase activity in yeast cells [27]. 

### 2.7. Electrophoretic Mobility Shift Assay (EMSA)

The full-length MaWRKY70 was inserted into the pGEX4T-1 vector to fuse to the glutathione S-transferase (GST) tag. GST-MaWRKY70 or empty GST (negative control) was expressed in Rosetta (DE3) *E. coli* cells, induced with isopropyl-D-thiogalatopyranoside (IPTG), and purified by the GST Purification Kit (Takara, Tokyo, Japan). Probes containing the W-box recognition sequence (C/T)TGAC(C/T)) from the target promoter regions were synthesized and biotin-labeled by 3′ biotin and HPLC purification [27]. EMSA was conducted with a LightShift Chemiluminescent EMSA Kit (Thermo, Waltham, MA, USA), and biotin-labeled DNA was observed on the ChemiDoc™ MP Imaging System (Bio-Rad, Hercules, CA, USA) [27].

### 2.8. Statistical Analysis

All experiments were performed at least in triplicate. Data were recorded as the mean ± standard error (S.E.) of three or six independent biological replicates. Significant differences between samples in cold-stored fruits and control fruits were determined by Student’s *t*-test (* *p* < 0.05 or ** *p* < 0.01). 

## 3. Results 

### 3.1. Physiological Changes during Banana Fruit CI Development 

**Figure 1:**
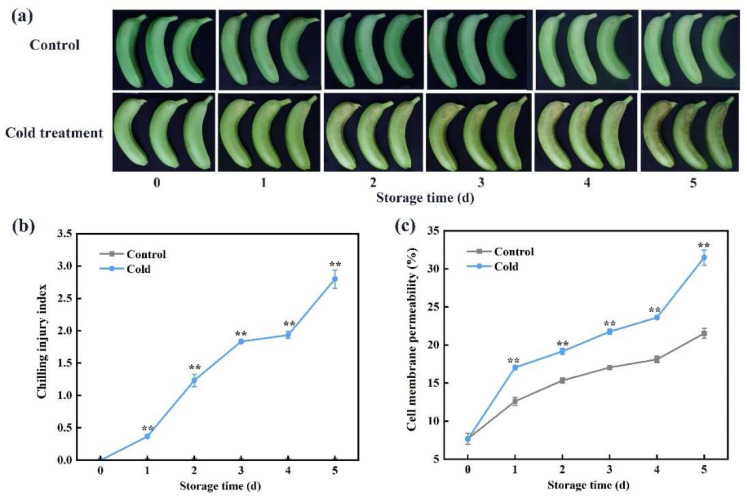
Cold storage induces chilling injury (CI) occurrence in banana fruit. (**a**) Changes in fruit appearance. (**b**) CI index and (**c**) cell membrane permeability in control and cold-stored bananas during storage. Each datum represents the mean ± standard error of three replicates, and the asterisks indicate significant differences by Student’s *t*-test (** *p*-value < 0.01).

Peel browning is an apparent symptom of CI in bananas. As displayed in Figure 1a, the fruits showed an intact peel appearance without any browning. All banana fruits did not exhibit any CI symptoms in the control group (22 °C), but fruit browning was seen after 1 day of storage at 7 °C, and it became increasingly severe (Figure 1a). The CI index and cell membrane permeability are used to evaluate the severity of CI progression [5]. In the control group, the CI index remained 0 during the whole storage period, but it increased greatly in the cold-stored group (Figure 1b). The cell membrane permeability displayed an increasing tendency with the extension of the storage time; it was increased remarkably in cold-stored bananas, and it maintained higher levels compared with control fruits during storage. In cold-stored bananas, the values of cell membrane permeability on day 5 were 4.13-fold higher than on day 0 (Figure 1c). 

### 3.2. Isolation of MaWRKY70 

**Figure 2:**
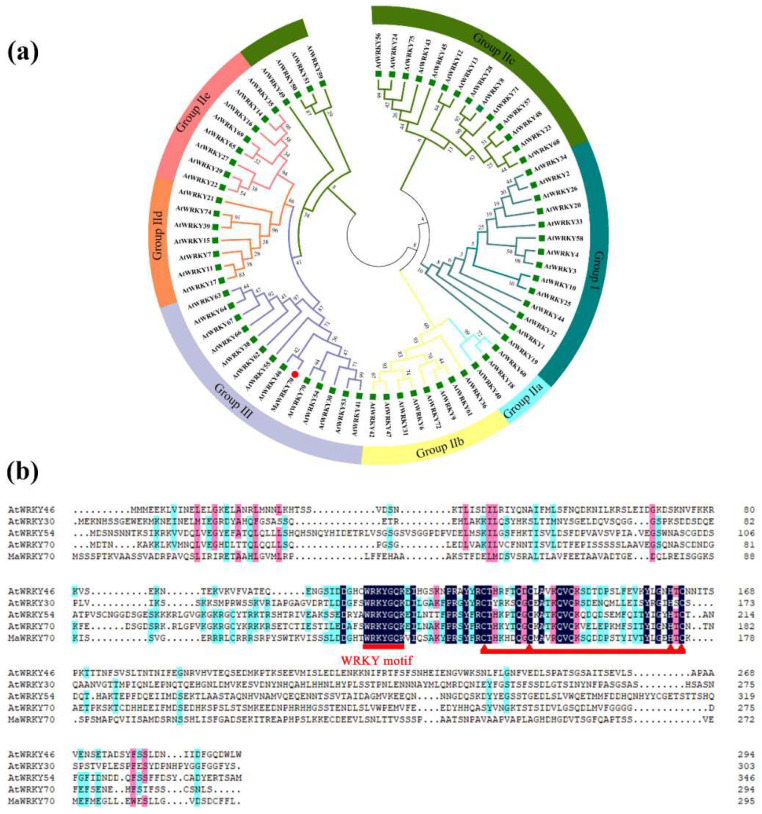
Sequence analysis of MaWRKY70. (**a**) Phylogenic tree of AtWRKYs and MaWRKY70. MaWRKY70, along with AtWRKY46, AtWRKY63, AtWRKY55, and AtWRKY70, was classified into Group III. (**b**) Multiple alignment of MaWRKY70 with AtWRKY46, AtWRKY54, AtWRKY55, and AtWRKY70. WRKY domain and C2HC zinc-finger motif are marked.

Banana WRKY TFs have been reported to play central roles in regulating CI development [17]. Based on our transcriptome database relevant to CI in banana fruits [2], a *WRKY* gene (Ma06_g17380), which was markedly upregulated by cold, was selected and identified. The open reading frame (ORF) of Ma06_g17380 is 888 bp, encoding a protein of 296 aa, and it was named MaWRKY70 (XP_009404676.1) following the NCBI database. To investigate the phylogeny of MaWRKY70, a phylogenetic tree showed that Ma06_g17380 was clustered with AtWRKY46 (AT2G46400), which belonged to Group III (Figure 2a). Moreover, the protein alignment of MaWRKY70 with AtWRKY46 (AT2G46400), AtWRKY54 (AT2G40750), AtWRKY55 (AT2G40740), and AtWRKY70 (AT3G56400) showed that they possessed a highly conserved WRKY domain and the putative C2HC zinc-finger motif (Figure 2b). 

### 3.3. Molecular Characterization of MaWRKY70

**Figure 3:**
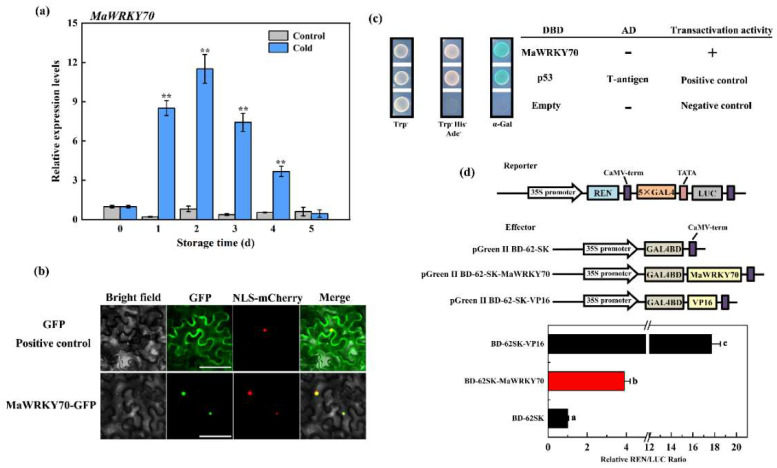
Molecular characterization of MaWRKY70. (**a**) Relative expression levels of MaWRKY70 in control and cold-stored banana fruit during storage, the asterisks indicate significant differences by Student’s *t*-test (** *p*-value < 0.01). (**b**) Nuclear localization of MaWRKY70 in tobacco leaves. GFP and mCherry signals were captured by a fluorescence microscope. Bars, 50 μm. (**c**) Transcriptional activation of MaWRKY70 in yeast cells. pGBKT7 and pGBKT7-53 + pGADT7-T were used as negative and positive control, respectively. (**d**) Transcriptional activation capacity of MaWRKY70 in tobacco cells. The ratio of LUC to REN indicates the transactivation ability of MaWRKY70. The ratio of LUC to REN of the BD-62SK vector was used as a calibrator (value set as 1). Each datum represents the mean ± standard error of three replicates, and the different letters indicate significant differences by Student’s *t*-test (*p*-value < 0.01).

To analyze the potential association between *MaWRKY70* and CI development in banana fruit, the gene expression (RT-qPCR) of *MaWRKY70* was studied during storage. The results showed that the expression of *MaWRKY70* was obviously upregulated in cold-stored banana fruit, but there was no obvious change in control fruit during the storage time (Figure 3a). To investigate the subcellular localization of MaWRKY70, it was fused with GFP and the MaWRKY70-GFP fusion protein was generated and injected into tobacco leaves for transient expression. As exhibited in Figure 3b, the GFP signal of the MaWRKY70-GFP fusion protein was observed in the nuclei of tobacco cells, while GFP fluorescence (positive control) was observed throughout the cell. NLS-mCherry was used as a marker, and it completely overlapped with the green fluorescence in the cell nucleus. 

To test the transactivation activity of MaWRKY70, we employed the Y2H assay in the yeast system and the DLR assay in tobacco leaves. For the Y2H assay, the yeast cells transformed with pGBKT7-53 + pGADT7 (positive control) and the yeast cells harboring pGBKT7-MaWRKY70 survived well on a selection medium (SD/-Trp-His-Ade) and turned blue in the presence of X-α-gal, but the negative control (pGADT7-T vector only) did not survive (Figure 3c), indicating that MaWRKY70 exhibited transcriptional activation activity in yeast. Furthermore, we studied the transcriptional activation of MaWRKY70 in tobacco leaves. Compared with BD-62SK negative control which was expressed as 1, BD-62SK-MaWRKY70 activities the LUC/REN ratio. Similar to the positive control, BD-62SK-VP16 enhanced this value (Figure 3d), suggesting the transcriptional activation activity of MaWRKY70 in tobacco leaves. Collectively, these results demonstrate that MaWRKY70 is a nuclear-localized transcriptional activator that is associated with CI development in postharvest banana fruit. 

### 3.4. Expression Patterns of MaLOXs during Banana Fruit CI Development

**Figure 4:**
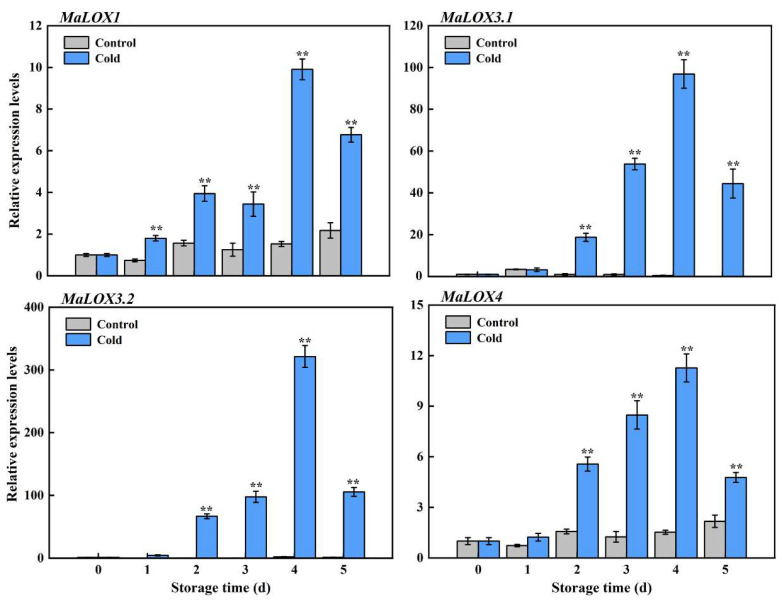
Relative expression levels of *MaLOX1*, *MaLOX3.1*, *MaLOX3.2*, and *MaLOX4* in control and cold-stored banana fruit during storage. Each datum represents the mean ± standard error of three replicates, and the asterisks indicate significant differences by Student’s *t*-test (** *p*-value < 0.01).

LOX promotes membrane lipids’ peroxidation, which has an important role in the CI development process. Our previous transcriptome revealed that the *MaLOX* genes were greatly upregulated by cold storage [2]; therefore, an RT-qPCR analysis was conducted to confirm the expression of *MaLOXs*. As shown in Figure 4, the expression of *MaLOX1.1* (Ma06_g26850), *MaLOX3.1* (Ma08_g23400), *MaLOX3.2* (Ma09_g15420), *MaLOX1.2* (Ma09_g19140), and *MaLOX4* (Ma10_g17560) was induced by cold treatment and they maintained higher levels in cold-stored bananas compared to control fruits. At the peak of the upward trend period (day 4 of storage), the mRNA transcripts of *MaLOX1*, *MaLOX3.1*, *MaLOX3.2*, and *MaLOX4* in cold-stored bananas were 3.29-, 3.57-, 5.05-, and 3.03-fold higher than those of control fruits (Figure 4).

### 3.5. MaWRKY70 Activates Transcription of MaLOXs

**Figure 5:**
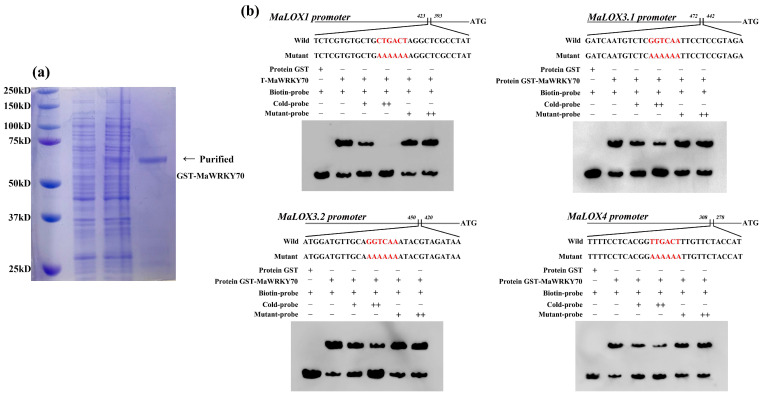
Electrophoretic mobility shift assay (EMSA) of MaWRKY70 binding to the promoters of *MaLOX1*, *MaLOX3.1*, *MaLOX3.2*, and *MaLOX4* containing W-box element. (**a**) Affinity purification of recombinant GST-MaWRKY70 protein as presented by SDS-PAGE gel stained with Coomassie brilliant blue. (**b**) EMSA assays. GST protein was used as negative control. GST-MaWRKY70 fusion protein binds to promoters of *MaLOX1*, *MaLOX3.1*, *MaLOX3.2*, and *MaLOX4*. Unlabeled probes (cold probe) with 50-fold (50×) and 500-fold (500×) were used for competition. Shifted bands suggest the formation of DNA–protein complexes. Symbols − and + represent absence or presence, respectively, and ++ indicates increasing amounts.

It is widely known that WRKY TFs regulate their target genes by binding to the W-box *cis*-element in the promoter [17]. Here, the promoter regions of *MaLOXs* were scanned, and we found that they all contained putative W-box motifs (Text S1). Moreover, the expression trends of *MaWRKY70*, *MaLOX1*, *MaLOX3.1*, *MaLOX3.2*, and *MaLOX4* were similar during the CI development process (Figure 3a and Figure 4). Therefore, we speculated that these four *MaLOXs* might be the targets of MaWRKY70. An EMSA was performed to verify the direct binding of the MaWRKY70 protein to the promoters of *MaLOX1*, *MaLOX3.1*, *MaLOX3.2*, and *MaLOX4.* An EMSA with the purified recombinant MaWRKY70 protein was performed to investigate whether MaWRKY70 could recognize the W-box. The results showed that the purified GST-MaWRKY70 fusion protein (Figure 5a) directly targeted the DNA biotin probes harboring the W-box cis-acting element in the promoters of *MaLOX1*, *MaLOX3.1*, *MaLOX3.2*, and *MaLOX4* and caused shifted bands, and the band shifting was weakened upon the addition of increasing amounts of the cold probe. No shifted band was observed when the GST protein alone was used with biotin probes (Figure 5b). These results demonstrate that MaWRKY70 targets the W-box motif in the promoters of *MaLOX1*, *MaLOX3.1*, *MaLOX3.2*, and *MaLOX4*.

**Figure 6:**
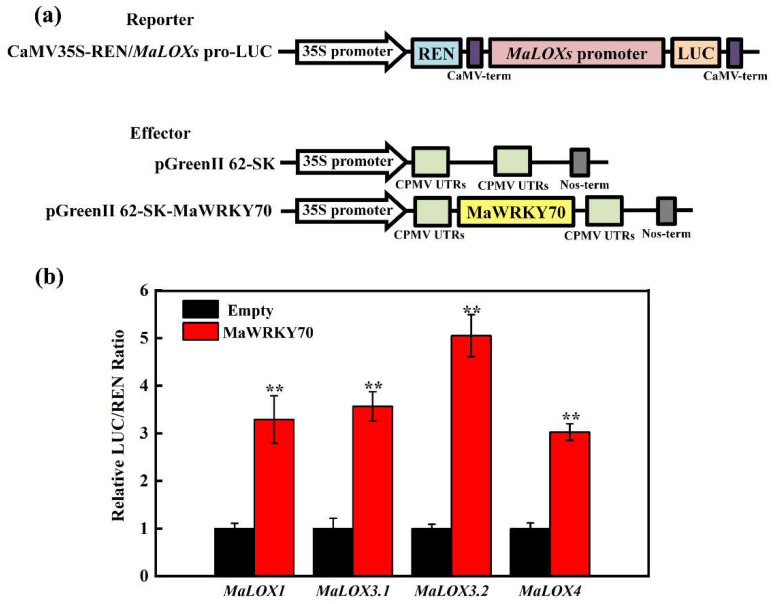
Dual luciferase reporter assay. (**a**) Diagrams of the reporter and effector vectors. (**b**) MaWRKY70 activated transcription of *MaLOX1*, *MaLOX3.1*, *MaLOX3.2*, and *MaLOX4*. The ratio of LUC/REN of the empty vector (62-SK) plus promoter was used as a calibrator (set as 1). Each datum represents the mean ± standard error of three replicates, and the asterisks indicate significant differences by Student’s *t*-test (** *p*-value < 0.01).

Since MaWRKY70 is a transcription activator (Figure 3c,d), the transactivation of *MaLOX1*, *MaLOX3.1*, *MaLOX3.2*, and *MaLOX4* by MaWRKY70 was further examined through the DLR system in tobacco leaves. For this experiment, MaWRKY70 driven by the 35S promoter was used as an effector, and the LUC gene driven by each promoter of *MaLOX1*, *MaLOX3.1*, *MaLOX3.2*, and *MaLOX4* was used as a reporter (Figure 6a). As displayed in Figure 6b, when 62SK-MaWRKY70 was co-transformed with the *MaLOX1*, *MaLOX3.1*, *MaLOX3.2*, or *MaLOX4* promoter, the LUC/REN ratio was obviously enhanced, as compared to the control, which involved co-transformation with the empty 62SK vector. MaWRKY70 significantly enhanced the reporter activity, 3.02-, 3.29-, 3.57-, 5.05-, and 3.03-fold, under the control of the *MaLOX1*, *MaLOX3.1*, *MaLOX3.2*, and *MaLOX4* promoters, respectively, compared with the empty 62SK vector (Figure 6b). These results indicate that MaWRKY70 is a transcriptional activator of *MaLOX1*, *MaLOX3.1*, *MaLOX3.2*, and *MaLOX4* and acts by directly targeting the binding motifs in their promoters.

## 4. Discussion

Cold storage is commercially applied in fruit preservation and transport to suppress respiration and metabolism so as to maintain the quality and prolong the shelf-life of fresh horticultural crops [26]. However, the banana fruit is susceptible to CI under low-temperature storage; the development of CI limits the transport and preservation of banana fruit during cold storage [28]. Therefore, a better understanding of CI development is important to improve the quality of postharvest banana fruit. A previous study reported that increased cell membrane permeability was consistent with the development of CI in pineapple fruit during cold storage [29]. Moreover, cold-stored mangoes showed higher levels of cell membrane permeability and more severe CI symptoms compared with control storage mangoes [30]. Here, the CI index and cell membrane permeability exhibited higher levels in cold-stored bananas as compared with control fruits (Figure 1).

The structural integrity of the cell membrane is related to the composition of the membrane lipids. Our previous work showed that membrane lipid degradation and peroxidation enzymes, such as PLD, PLC, DGK, PLA, lipase, and LOX, were increased in line with the CI development process, and the gene expression of *MaLOXs* from the transcriptome was significantly induced by cold [27]. Similarity, the increased activity and expression of PLD, LOX, and lipase in ‘Nanguo’ pear accelerated membrane lipid degradation and peroxidation, resulting in severe browning and CI [31]. In cucumber fruit, cold stress also induced the expression levels of the *CsPLD* and *CsLOX* genes [10]. CI development was in parallel with the increased PLD and LOX activity and the upregulated expression of *CmPLD-β* and *CmLOX* in cold-stored Hami melon fruits [32]. The upregulated expression of *LOX*, *PLD* and lipase and their enhanced activity were found in cold-stored peaches [33]. *CaPLDα4* was associated with CI development in sweet pepper during cold preservation [34]. Here, RT-qPCR assays were conducted to evaluate the expression levels of *MaLOXs*. Compared to control storage, chilling storage obviously promoted the expression of *MaLOX1*, *MaLOX3.1*, *MaLOX3.2*, and *MaLOX4* (Figure 4), implying the occurrence of membrane lipid injury and CI in cold storage. 

The WRKY family members are widely involved in regulating various biological processes in plants, including fruit ripening, leaf senescence, plant hormone signal transduction, and the biological and environmental stress responses [35]. Fifty-eight WRKY proteins in eggplants were identified and the transcriptome showed that the *WRKY* genes were differentially expressed in response to a cold environment [36]. CsWRKY46 confers cold tolerance by modulating cold-stress-responsive genes in cucumber [37]. A cold-induced WRKY gene in grape, *VvWRKY28*, regulated the expression of cold-stress-related genes and played a role in cold stress tolerance [38]. In this study, a CI-inducible WRKY TF, MaWRKY70, was identified. Subcellular localization and transactivation analyses showed that MaWRKY70 was nuclear-localized and displayed a transactivation ability. Moreover, the multiple sequence alignment and phylogenetic tree analyses of MaWRKY70 revealed that it was a homologue of AtWRKY46, AtWRKY70, and AtWRKY54 (Figure 2). In Arabidopsis, AtWRKY46 was found to be a MAPK phosphorylation target involved in plant defense responses [39]. AtWRKY70 could negatively regulate root elongation by inhibiting ROS production [40]. AtWRKY54 interacted with both AvrRps4 and PopP2 and was involved in cell death [41]. These findings suggest the similar stress response function of MaWRKY70.

**Figure 7:**
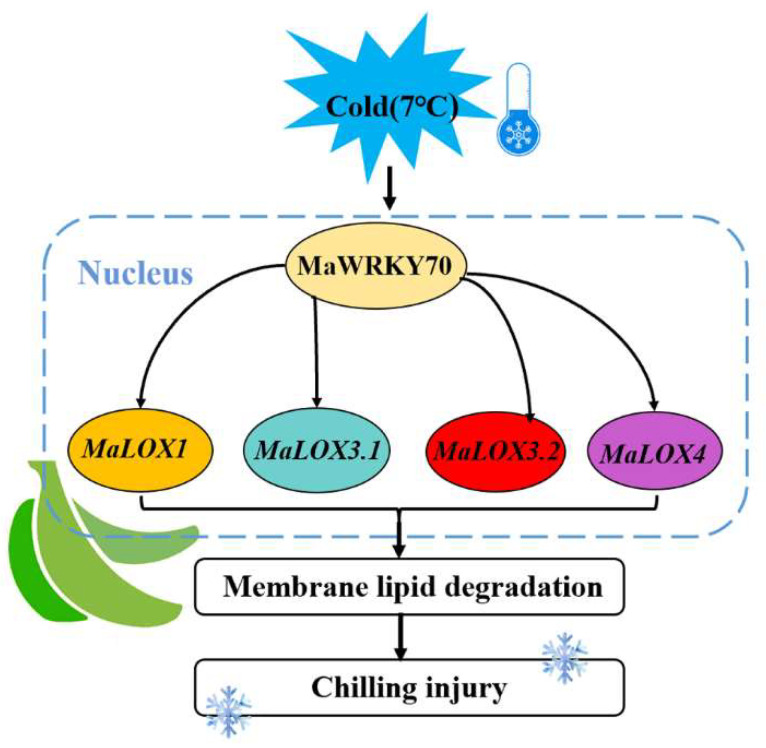
Working model illustrating the regulation of CI development by MaWRKY70. MaWRKY70, which was induced by cold, directly promoted the expression of *LOXs* (*MaLOX1*, *MaLOX3.1*, *MaLOX3.2*, and *MaLOX4*) to accelerate membrane lipid degradation, ultimately leading to CI development.

WRKY TFs could activate or inhibit the transcription of target genes by specifically recognizing the W-box binding elements in the downstream target gene promoter region, and they then participate in plant development and the stress response [42]. For example, FvWRKY50 promoted the expression of *FvCHI* and *FvDFR* by binding to their promoters in strawberry fruit [22]. In banana fruit, *MaWRKY31*, *MaWRKY33*, *MaWRKY60*, and *MaWRKY71* were involved in abscisic acid (ABA)-induced cold tolerance via binding to the W-box motifs in ABA biosynthesis genes’ (*MaNCED1* and *MaNCED2*) promoters and promoted their expression [43]. In wheat, TaWRKY19 activated the transcription of *DREB2A*, *RD29A*, *RD29B*, and *Cor6.6* and bound to their promoters to enhance their abiotic stress tolerance [44]. In apple fruit, MdWRKY40 was directly bound to the promoter of *MdDFR* to promote anthocyanin accumulation, and it also interacted with MdMYB15L to modulate its inhibitory effect on MdCBF2, indirectly increasing the expression of *MdCBF2* and enhancing the plant’s cold resistance [45]. Similarly, W-box elements were found in the promoter regions of *MaLOX1*, *MaLOX3.1*, *MaLOX3.2*, and *MaLOX4* (Text S1). Therefore, an EMSA and DLR were performed, and the results demonstrated that MaWRKY71 directly bound to the four *LOX* promoters (*MaLOX1*, *MaLOX3.1*, *MaLOX3.2*, and *MaLOX4*) and activated their expression (Figure 5 and Figure 6). Our findings suggest that a banana fruit WRKY TF acts as a positive modulator of CI development, which is associated with their involvement in regulating membrane lipid peroxidation by activating *LOX* expression. A working model of the possible role of MaWRKY70 in CI development in banana fruit is proposed (Figure 7). During cold storage, the expression level of *MaWRKY70* is increased, and it directly activates the transcription of *LOXs* (*MaLOX1*, MaLOX3.1, *MaLOX3.2*, and *MaLOX4*). Enhanced LOX activity leads to membrane lipid degradation and to an apparent CI symptom. Collectively, our findings facilitate advances in understanding the molecular events governing CI development in cold-sensitive fresh fruit.

## 5. Conclusions

In summary, the results show that cold stress can cause CI in banana fruit during the preservation period, as evidenced by the increasing CI index and cell membrane permeability. More importantly, a postharvest CI-inducible transcriptional activator, MaWRKY70, from banana fruit was identified. MaWRKY70 binds to the promoters of four *LOX* genes, namely *MaLOX1*, *MaLOX3.1*, *MaLOX3.2*, and *MaLOX4*, and activates their transcription. This ultimately accelerates the peroxidation of membrane lipids, resulting in membrane structure damage and peel browning. Although our findings illustrate the transcription-regulatory role of WRKYs in CI development in postharvest banana fruit, the post-translational regulatory module that causes CI in banana needs further investigation.

## Data Availability

The original contributions presented in the study are included in the article/Supplementary Materials, further inquiries can be directed to the corresponding author.

## Supplementary Materials

The following supporting information can be downloaded at: https://www.mdpi.com/article/10.3390/foods13060854/s1, Table S1: Summary of primers used in this study; Text S1: The *cis*-elements of WRKY TFs in the nucleotide sequences of *MaLOX1*, *MaLOX1-2*, *MaLOX3.1*, *MaLOX3.2* and *MaLOX4* promoters. *cis*-elements of WRKY TFs were underlined. The probe nucleotide sequence used in EMSA assay is indicated in bold. Translation start site (ATG) was shown in yellow.
