# Supplementary material for: The Role of MaWRKY70 in Regulating Lipoxygenase Gene Transcription during Chilling Injury Development in Banana Fruit
Source: Foods. 2024 Mar 11;13(6):854. doi: 10.3390/foods13060854 (PMC10969808; doi:10.3390/foods13060854)
Supplement: Supplementary file 1 [file foods-13-00854-s001.zip › foods-2836821-supplementary.pdf]

Table S1. Summary of primers used in this study.

Assay	Primer sequence (5'-3')	Restriction Site
**Full length cloning**	*MaWRKY70-F:* ATGTCGTCGTCCCCCACG<br>*MaWRKY70-R:* CTACAGGAAGAAACAATCAGAGTC	
**Subcellular localization**	*MaWRKY70- GFP-F:* ATCTAGAGCAGTCGACGGTACCATGTCGTCGTCCCCCACG<br>*MaWRKY70- GFP -R:* CTCCTCGCCCTTGCTCACCATCAGGAAGAAACAATCAGAGTC	*Bam*H I<br>*Kpn* I
**RT-qPCR**	MaEIF5A-*qF:* ACACCAAAGATGATCTGAGGCT<br>*MaEIF5A-qR:* AAGGAACACCAGGGCAACAGT<br>*MaWRKY70-qF:* CTCAGGTTATCATCTCAGCC<br>*MaWRKY70-qR:* TTTTGAGAGAAGGATGTGGG<br>*MaLOX1-qF:* CATACGCCACGAGGACTCTGTT<br>*MaLOX1-qR:* TGCCGAGTACACTTTGCTTACA<br>*MaLOX1-2-qF:* AGGAGTTTGCTCGGGAGATGCT<br>*MaLOX1-2-qR:* CTCGATGTGGGCTGCTGTGATT<br>*MaLOX3.1-qF:* CGACCGAGCGACCTCCTCTTCT<br>*MaLOX3.1-qR:* GTCACAGCCGCCTGGACCTTAA<br>*MaLOX3.2-qF:* TGGCAGGCATCAATCCCGTTAA<br>*MaLOX3.2-qR:* GGTGCGAGTGCCGTAGGCTTTT<br>*MaLOX4-qF:* ACCAAGCGAATCTTCTTCAGCA<br>*MaLOX4-qR:* CCAAGTCATTGTAGGTGGCGTA	
**Promoter isolation**	*MaLOX1 pro-F:* CTATGAGTCAAGTAACAA<br>*MaLOX1 pro-R:* CATCTTCGGTTCTCTTTTTCCT<br>*MaLOX1-2 pro-F:* ATGAAATCTAAGGCAAACC<br>*MaLOX1-2 pro-R:* CATCGTTGTCTCTACCGTAATGG<br>*MaLOX3.1 pro-F:* TGCTCCTACCATAAGTTCC<br>*MaLOX3.1 pro-R:* CATGGAAGCGAGCGAGTG<br>*MaLOX3.2 pro-F:* CATACCTACGCAACAGCTA<br>*MaLOX3.2 pro-R:* CATCCAATATCGTCCCCTTCC<br>*MaLOX4 pro-F:* GATGCGTCCTTTGGT<br>*MaLOX4 pro-R:* CATTCCCTCCCTTCTTTTCT	
**Y2H assay**	*MaWRKY70-pGBKT7-F:* ATGGCCATGGAGGCCGAATTCATGTCGTCGTCCCCCACG<br>*MaWRKY70-pGBKT7-R:* TGCGGCCGCTGCAGGTCGACGCAGGAAGAAACAATCAGAGTC	*Eco*R I<br>*Sal* I

**EMSA assay**	*MaWRKY70-pGEX-F:* GTTCCGCGTGGATCCCCGGAATTCATGTCGTCGTCCCCCACG	*Eco*R I
	MaWRKY70-pGEX-R: TCAGTCACGATGCGGCCGCTCGAGCTACAGGAAGAAACAATCAGAGTC	*Xho* I
	MaLOX1 probe-F: TGGAGTCTATAGAGTCAAAAGATAATATCA	
	MaLOX1 probe-R: TGATATTATCTTTTGACTCTATAGACTCCA	
	MaLOX1-2 probe-F: TCTCGTGTGCTGCTGACTAGGCTCGCCTAT	
	MaLOX1-2 probe-R: ATAGGCGAGCCTAGTCAGCAGCACACGAGA	
	MaLOX3.1 probe-F: GATCAATGTCTCGGTCAATTCCTCCGTAGA	
	MaLOX3.1 probe-R: TCTACGGAGGAATTGACCGAGACATTGATC	
	MaLOX3.2 probe-F: ATGGATGTTGCAGGTCAAATACGTAGATAA	
	MaLOX3.2 probe-R: TTATCTACGTATTTGACCTGCAACATCCAT	
	MaLOX4 probe-F: TTTTCCTCACGGTTGACTTTGTTCTACCAT	
	MaLOX4 probe-R: ATGGTAGAACAAAGTCAACCGTGAGGAAAA	
**Dual-luciferase transient expression assay**	*MaWRKY70-BD-62SK-F:* CGCCGTCTAGAACTAGTGGATCCATGTCGTCGTCCCCCACG	*Bam*H I
	MaWRKY70-BD-62SK-R: TCGATAAGCTTGATATCGAATTCCTACAGGAAGAAACAATCAGAGTC	*Eco*R I
	MaWRKY70-62SK-F: GGCCGCTCTAGAACTAGTGGATCCATGTCGTCGTCCCCCACG	*Bam*H I
	MaWRKY70-62SK-R: ATCGATAAGCTTGATATCGAATTCCAGGAAGAAACAATCAGAGTC	*Hind* III
	MaLOX1pro-LUC-F: CACTATAGGGCGAATTGGGTACCCTATGAGTCAAGTAA	*Bam*H I
	MaLOX1pro-LUC-R: TTTATGTTTTTGGCGTCTTCCATCATCTTCGGTTCTCTTTTTCCT	*Hind* III
	MaLOX1-2pro-LUC-F: CACTATAGGGCGAATTGGGTACCATGAAATCTAAGGCAAACC	*Bam*H I
	MaLOX1-2pro-LUC-R: TTTATGTTTTTGGCGTCTTCCATCATCGTTGTCTCTACCGTAATGG	*Hind* III
	MaLOX3.1pro-LUC-F: CACTATAGGGCGAATTGGGTACCTGCTCCTACCATAAGT	*Bam*H I
	MaLOX3.1pro-LUC-R: TTTATGTTTTTGGCGTCTTCCATCATGGAAGCGAGCGAGTG	*Hind* III
	MaLOX3.2pro-LUC-F: CACTATAGGGCGAATTGGGTACCCATACCTACGCAACAGCTA	*Bam*H I
	MaLOX3.2pro-LUC-R: TTTATGTTTTTGGCGTCTTCCATCATCCAATATCGTCCCCTTCC	*Hind* III
	MaLOX4pro-LUC-F: CACTATAGGGCGAATTGGGTACCGATGCGTCCTTTGGT	*Bam*H I
	MaLOX4pro-LUC-R: TTTATGTTTTTGGCGTCTTCCATCATTCCCTCCCTTCTTTTCT	*Hind* III

Supplementary Text S1. The *cis*-elements of WRKY TFs in the nucleotide sequences of *MaLOX1, MaLOX1-2, MaLOX3.1, MaLOX3.2* and *MaLOX4* promoters. *cis*-elements of WRKY TFs were underlined. The probe nucleotide sequence used in EMSA assay is indicated in bold. Translation start site (ATG) was shown in yellow.

> *MaLOX1* (Ma06_t26850)

CTATGAGTCAAGTAACAAGCAGTTTGTTGAATGAGGAGTTGAGAAGAAAGATTTCGGCAACATCTCAGAATAATTCACAGGCACTTA

TCTTAGAGAACAAAGGAAGGTCAAAGTCTAGAAGCAGTTCACGCATAAGTAGAAGCAAGTCAAGATCAAGAAAAGATATTATTTGC

TATAATTGTAGTGAGAAAGGATATTACAAGAACCAATGTAAGCAACCTAAGAAGAGCAAGAAAAAGGGAAAAAAAG**TGGAGTCTA**

TAGAGTCAAAAGATAATATCAACTAGACCTAAGAGGTATACACCACGGGGCAAAATCAATTAAATGAAATAACAATCAAATAAAT

ATCAAGATATATGTGGAAAACCCCTCCAATGAAGGGTAAAAACCACGGGGCAAACTAGAGATAATCCACTATGAGAATAATGAATA

TACAAATCTCAATCTCTTACCCTAAACCCTAGCAACAATCACAAGAGAATAACTAGGATACAAGGATCACATCACTGCCTACAATAA

TTGAGAACGTAGCCACCACACCCCTCTTAATGTTAATTAGTGTTAGGTTAAGAGGGGGTGAGCTATGGGCTGCCCAAGCCTACTATG

GGCTGAATTTGTGGACTGCCAATCTAAGAATATGAGCCTAAGAATTTAATAAATTTAAATATGCAAACAATTTTGTACCATTAAAGC

ATGTAGATTATTTTTATGTCTTAAAAGAGCATGGTTTCAATGCAACATATGTGCATTAAGACAATCCAGACAAATGTAATGCACAATG

TATTAAGACAAAAAAATCATCTAGATGATAAGTAATTTTTCTACTAATTTAGAAATGTAGTTAAAAGTCTAAGTTAACATAACACTTA

CTAAAATCGCTGCAATTAGTCATTTATAAAACTAAGTCTAAGTATTTGTTTGTATCATTGTTATATTTATTTTGTGTGGAAAAGATATACA

ACTAAGTCTCGCGCAGATGACACATAAATCAGAGTTCACATTGGACCGCGATTGGCGGCCATGTGAAACGCATGGATGCTCAATTATTT

GCGTTATCCGCACATTGAAACAGTGATCAGGGAGTGGGTCCCGACAAAACACCTATTTAATTCAGTGGACCCAACCACGTAAAATTATT

TGGGGAAGCATTTATGGACACGAGAAACCCGTGCTTGTTCGACACGCAGGCGGATGCTCTGCATCAATAAATACCACCTCCGCTGCAA

TCGATCTCACTGCAACGTTCACTCTCTTAGCTATTAGTCGTCGCTATCCACTGTGTTACAGGAAAAAGAGAACCGAAGATG

> *MaLOX1-2* (Ma09_t19140)

ATGAAATCTAAGGCAAACCCTAATTCTTGAGATTGATTGTTTTTCTTCAAGTGATGAACTTATAATAATAATTAACAGGGAAAAAGAC

TCAAGAGTCACGTAAAAGTGTTTCGAGGTTAAATTTAGATTATACCTTTTAACGCCTTCTTCTTTCCCCCTTTTAAGCTCGCTCTCAAA

CCACTATTTTGTTTTATTTTGTTGACTTGTACATCATCTCTTCTCCCTCAAGCTTTTATTGTGAACGTCACAAATGAACCCTATCGCATG

CCTTCGGCATGTTTACAAGAAAAGCACTATTCTACTCACACTCCAAACTCTTTTTTTTTTTTTTTGCCATTCGTATAAAAACAAGGGTT

AGATGAAGATTTGTCCGATCCAACTCGATGAGAAAGACAATCAGACCTGTGTCACCATCGAATTATTAATAATTTTTTTCTTAATATT

CTATAGATAACTTAAGGATATCTACAAGATTTCGTCCAATATGGAGCCCACGCTTGTCTTGTCGAACGCATCATCACT**TCTCGTGTG**

CTGCTGACTAGGCTCGCCTATCAATTTGGACTGAAACATGAGGGTCCAACTTGGAATTACATACAAACGATCATATTTGTCGAACC

GAATAGTATTATTTATTATACATGTCAACATGCCTACTACCAATGGTTACATACCTCAAAGAAACATAAAGTAGAGAGAAAGATGAA

AAGGAGACCATCACGTGATATAACGAGGGAAAGAAAAAAAAAAAAACAAAAACAAAAGAGGGCCATCAAACAGACATTCTCCCCG

AGGGAGAGGAAAGTGCCAAGAAGATGATGAACGCTCCACCTCCATGCTATATACATCCCCTACTCCATCCCTAACCCTCCCCACCAT

TGACTTCTCCTCCTCTTTCAGTCCTCCCTTCTGCTTCCTTAGCCCCTCCCATTACGGTAGAGACAACGATG

> *MaLOX3.1* (Ma08_t23400)

TGCTCCTACCATAAGTTCCAAACGAGTTGGAAGTATGTACGTGTTTGGATTCTAAGATGATTCCCTTTTGGCAAAAAATGGATGGGAT

CCGATCAAAATTAATATTGATAAATATTTATATATTTTTTATTATTAAATAAATCACCTTTGCATAAATGGTAGAAAATGATATAAAT

ATTCATTTTCCCATTGGCGTCGGATTGGGCTGTGCTCTTATTCCGGTCAACGCGGATCTCCGTCCAAATTAACGAATGGCTTCGAGCG

CAAAGCATTTGCACGTTAAACAGGCGGCTAATCAACGACCTCAAACTTCCATGCCCATTCTACCCTCCCTTTCCCCCATAATGACACG

ACATCAAACAGAGGCAACCAATGGTGGAT**GATCAATGTCTCGGTCAATTCCTCCGTAGA**CAGTTGCCAAAGGGATAATTCTTTCTT

AGTATCAGTCGTAAAATATTCTTTGAATTGACAAAAAAGCCACATTTTATATGTCTTTATGTATTAGTCGTAGAGGGACCGAATTGGT

ATTTTGTTCTCAGCGAGGGCATCGGCGGTCATTTTAACCGATTACAACGTCCATCATTGCCGCGAAGCAGCCCATCTCGCCTTCTTTAT

GGCAACCGGGAAAAACGTGTGGCCGGCGCCCTAAAAGCGCAAGGATTTCAAGCCCCTGTTTTACCACACCCCCCTCCCCCCCTTCTA

TCGTCCTATAAGAAGCAACGACTCATCCCTCTTCTCCCCCCAAAAGGAAGGATAGAAGACCCCCAGCCCTCCTCTGCTTCTCCAAATT

CCCTTTGTTTTCGTAACCAAGCTCGAGTTTGGAGATAGACGAAGACGCACTCGCTCGCTTCCATG

> *MaLOX3.2* (Ma09_t15420)

CATACCTACGCAACAGCTAATAAGTTCGATACACGTGGCTTCCTATTCCCTTAGCTTTGGAATCATGTGGGTTATTATGCCCTTTATAA

ACCCACCCAAATTGTTGTCCTCCCATCGGTTATTTGATATCCATCAATTAATTATTATCATTATGACGTCGGAATCCCATCCGAGCGTC

GGAGGACGGTGTGGCGAATTGGGCTGCGTTACATACGTTTAGGTGTTTGACCACACGTGTGGCGGATGTGGGTCAACACGGATCTCC

GTTCAAAGTGAAGGAATCGCACGAAGGCAAAAACCATTCGCACGTGGAGCAGGTGGGGGCGCCCTGCCCTATTCATTCGGACCCCA

CCAAGTTCGCATGCCCTCAATTCTTTCACCATATGACAGTGGTCTCCCCCATTAACTCAGTGGGTGAGAGGCGACGTGGCCACCATCA

CACGCTCCCAAAGCTACTTTGTTATGTATACTTTTTCCTCTTTTTCTTGGCCACGTCAGCACGGTGTGAATAACGAGTTACTTGTGCGG

AACACCAGCAATGATTGATTACTGCTTACGTGGAATATTCAGGAATATCTGGCAATTTCACCTACTTGGCATCAGATTGAATCCGTTT

GGGTTCGGTTCGAAATCATTCTACTCGATTCAATTTTGATTTTGGACCGGTTCAATTTCGGTTCAAGCCGTGGGGGGTCACTGGGATT

CCTCCGCGAAGAGCACACATGTTTTTTGCCAGCACGCGTGTTGCGAAGAACACGACCGCATCACATACACTGTAATCGTTTCCAGCA

CCTGAACGCACTCGCCTTCCCCCTTCCTTTGCTTCACCATTT**ATGGATGTTGCAGGTCAAATACGTAGATAA**ATAAGCTAAGATTTT

TCCGAGCTAAATAAATCATATTATCCTTTTACACTTGGGATAATGGAAACCCATACGATTTCTACATGTTCTGTTTGTGTTCCGTCATG

CTTACGGGACTGGATTGGTATTTAAGGATTTGTGGTGGGCATCGGCGGTCCTTTGAAACGGACGTCGTTGCCGCGAAGCTGCCATTTG

GCCCTTATTTATCGCGAGGGAGAAAGCGTGTCCGTGTTGCAATGCCCATTTTACCCACTCGCCAGCCCCTATATAGTAGGACTTGCCC

ACCTTCCCCTGTTCGACCAAGGAGAGTCACCAAAGAAGGGGAGGAATTCAAGCGGACAGGAAACCAAGTCTTGCGCTTCTGCCCAA

AAATCTACTGTTTCCATCGGGAAGTCGAGATTTTGGGAAGGGGACGATATTGGATG

> *MaLOX4* (Ma10_t17560)

GATGCGTCCTTTGGTTAAGCTTTTATTATTTGTGATAGCTATGCATCGACGAAGATAAAGATGAATATCCTCGTTTCACTTTCAATTTC

ATATTTTCAAACTCTACTCACGAAATTAACGATCACTCGTTGTCATACAAGAAATGTATGAAAAAGGATTAGAGTGCATAGTCTTCTC

CCTTTAAATCGAAGATTTGGGTGACAACAACGGGAAGCTACTTAGGCGAGTCATTAAAGAATTAGACTTCCGATGACTTCAATTATT

ATAGAGAAATTTCGGTATTTAAATGATCGAAAAGAAGATAGTAAGTGGCATTTACACCACTCATTAGTTTGTGGTTGAGATAATAAA

TGATAAGTTTTATGTCATAAAAAATAACACCACGAATTCGTTTTTTTACTTGAACGTCACGAGATCGCCATGTCCCTTTATTGGAGAA

ATTGACTTGGCAGATCGAACTCAACAATCACACCAAGCAAGATTGAATTCTATTGATGTTGTCCTGTGAGCAACTAAAAACATGATA

GCAAGAATCAAACAAACAAGTTTGATATCTCCTCCTGTCCTTATGAAGTACCTACGATGAGGAAATCATCCCCATCATGTTCCAATGA

TCGAGTTGCGGGATTCAGTGTGAAAGTAGTACTGCTTCTGCCATTGATATGCCATTTGAACACCACAGATGGGCATGCCGGTCCTTCG

AGCTGCAGAAAATGAACCCCACTACCTGGAAACATCCTTC**TTTTCCTCACGGTTGACTTTGTTCTACCAT**CACGCAATCGATCACA

CCCGTACAGCATGTGTTTATGAGCTATAAACGCCAAGAAATCAAACAAGAAACGTGCACGAGGTAGAAAAGTAGAAACCAAGGGGC

CGCCTGCCGCCCCTCCCTTCTTATATTTGTCAACCCATCCTTAGATCACACCGCAATAATTCTTGTCGCAACAAAACAGAGCAGGAAG

AAGAAGAAAGCTCCCCCCCCCCCTTCTTTCTCCTGAAACTTTGCTCGAGTGACTACTTTGATCTTTAGGAAGAAAAGAAGGGAGGGA

ATG
